# Deep Venous Thrombosis and Bilateral Pulmonary Embolism Revealing Silent Celiac Disease: Case Report and Review of the Literature

**DOI:** 10.1155/2017/5236918

**Published:** 2017-12-12

**Authors:** Igor Dumic, Scott Martin, Nadim Salfiti, Robert Watson, Tamara Alempijevic

**Affiliations:** ^1^Department of Hospital Medicine, Mayo Clinic Health System, Eau Claire, WI, USA; ^2^Mayo Clinic College of Medicine and Science, Rochester, MN, USA; ^3^Department of Pathology, Mayo Clinic Health System, Eau Claire, WI, USA; ^4^Department of Gastroenterology and Hepatology, Mayo Clinic Health System, Eau Claire, WI, USA; ^5^Department of Family Medicine, Mayo Clinic Health System, Eau Claire, WI, USA; ^6^Department of Gastroenterology and Hepatology, Clinical Center of Serbia, Belgrade, Serbia; ^7^School of Medicine, Belgrade University, Belgrade, Serbia

## Abstract

Celiac disease (CD) is a systemic, chronic autoimmune disease that occurs in genetically predisposed individuals following dietary gluten exposure. CD can present with a wide range of gastrointestinal and extraintestinal manifestations and requires lifelong adherence to a gluten-free diet [GFD]. Venous thromboembolism (VTE) as a presentation of celiac disease is unusual and rarely reported. We present a case of a 46-year-old man who was admitted for shortness of breath and pleuritic chest pain and was found to have iron deficiency anemia, deep venous thrombosis, and bilateral pulmonary emboli (PE). After work-up for his anemia, the patient was diagnosed with CD. Comprehensive investigation for inherited or acquired prothrombotic disorders was negative. It is becoming increasingly recognized that CD is associated with an increased risk for VTE. PE, however, as a presentation of CD is exceedingly rare and to the best of our knowledge this is the third case report of such an occurrence and the only case report of a patient from North America. It is important to recognize that the first symptoms or signs of celiac disease might be extraintestinal. Furthermore, VTE as a presentation of CD is rare but life-threatening.

## 1. Introduction

Celiac disease (CD), or gluten sensitive enteropathy, is a common, systemic autoimmune disease that occurs in genetically predisposed individuals secondary to exposure to dietary protein gluten and requires lifelong dietary treatment [1]. The prevalence of celiac disease worldwide is about 1% with wide geographical variations. It affects about 0.3% of the population in Germany and up to 2.4% in Finland for reasons that are not completely understood [2, 3]. The classic form of CD manifests as a malabsorption syndrome associated with chronic diarrhea, mineral deficiencies, failure to thrive, and weight loss. Not all patients with celiac disease have these classic symptoms. In fact, many patients who suffer from celiac disease have only extraintestinal manifestations or even no symptoms at all; these forms are termed atypical and silent celiac disease, respectively [4].

Hematologic abnormalities are frequently found in CD patients, with iron deficiency anemia (IDA) being the most common. Apart from IDA, other hematologic abnormalities seen in patients with CD are thrombocytosis, splenic hypofunction, leukopenia, IgA deficiency, enteropathy-associated T cell lymphoma (EATL), and rarely venous thromboembolism (VTE), including deep venous thrombosis (DVT) and pulmonary embolism (PE). Osteoporosis, elevated transaminases, aphthous stomatitis, and chronic fatigue are other nonhematologic extra intestinal manifestations of CD [4–6]. PE has rarely been reported as a presentation of celiac disease [7, 8]. Here we report a case of pulmonary embolism that was the presenting feature of celiac disease and review of the literature.

## 2. Case Presentation

A 48-year-old Caucasian man from Wisconsin, USA, was referred to our hospital for further evaluation of iron deficiency anemia, palpitations, dizziness, and right calf discomfort. His symptoms progressively developed over a period of two weeks before presentation. Apart from a history of intermittent heartburn relieved by calcium carbonate, he had no other major medical problems and was not on any prescription medications. He was not a smoker and did not drink alcohol or use any illicit drugs. He denied a previous history of malignancy or venous thromboembolism. His parents and siblings were healthy and there was no history of VTE in any first-degree relatives. He was complaining of mild dizziness and intermittent mild shortness of breath that started on the day of presentation. He denied any chest pain, syncope, bloody stools, or fevers.

Physical exam revealed a well appearing male in no distress. He was afebrile, normotensive, and tachycardic with a heart rate of 103 beats per minute. Laboratory studies showed severe iron deficiency anemia (Hgb: 91 g/dL, MCV: 60 fL, iron: 14 mcg/dL, iron saturation: 4%, TIBC: 369 mcg/dL, and ferritin: 7 mcg/dL). Other laboratory studies included WBC: 5.8, Plt: 269, and INR: 1.1. Fecal occult blood testing was negative. He had no microscopic hematuria and liver function tests were normal. Troponin I was undetectable.

**Figure 1:**
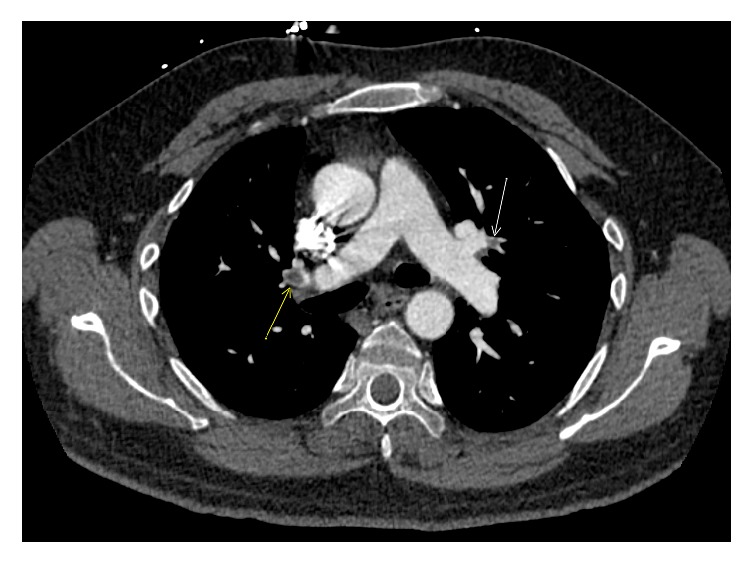
Axial CT image of the chest at the level of the main pulmonary artery bifurcation. Arrows denote two of the multiple pulmonary artery emboli (filling defects) which were observed bilaterally in this patient within both lobar and segmental pulmonary arteries.

Doppler of the right lower extremity showed extensive DVT, occlusive in the calf veins, popliteal and lower segments of right femoral vein and nonocclusive thrombosis in the mid and upper segments of the femoral vein. CT scan of the chest with IV contrast showed bilateral pulmonary emboli, greater on the right, most prominently seen within the distal right main pulmonary artery and to a lesser degree on the left within segmental and subsegmental branches (Figure 1). There was some flattening of the interventricular septum suggesting right heart strain. Echocardiogram was subsequently performed and demonstrated normal left ventricular ejection function, mild decrease in right ventricular ejection function, and no wall motion abnormalities. He received IV heparin initially and was transitioned to apixaban for long term anticoagulation. Extensive work-up for hypercoagulability revealed no abnormalities. It included Factor V Leiden, protein C and S levels, prothrombin mutation, antithrombin deficiency, antiphospholipid antibodies, and homocysteine levels. CT scan of abdomen and pelvis with and without IV contrast showed no masses suspicious for malignancy.

**Figure 2:**
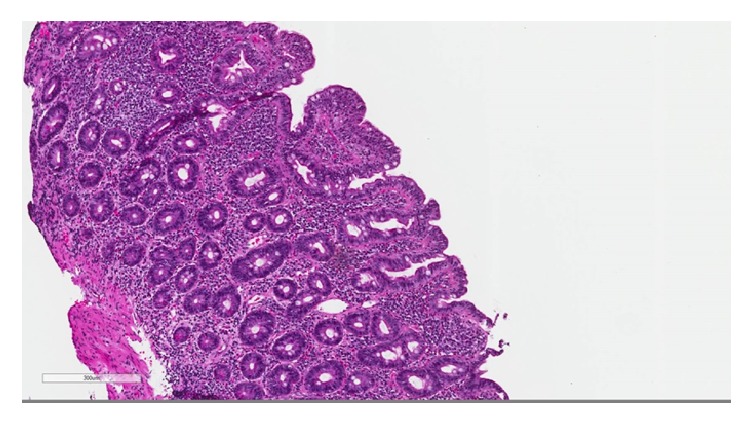
Duodenal biopsy demonstrating partial villous atrophy with crypt hyperplasia.

**Figure 3:**
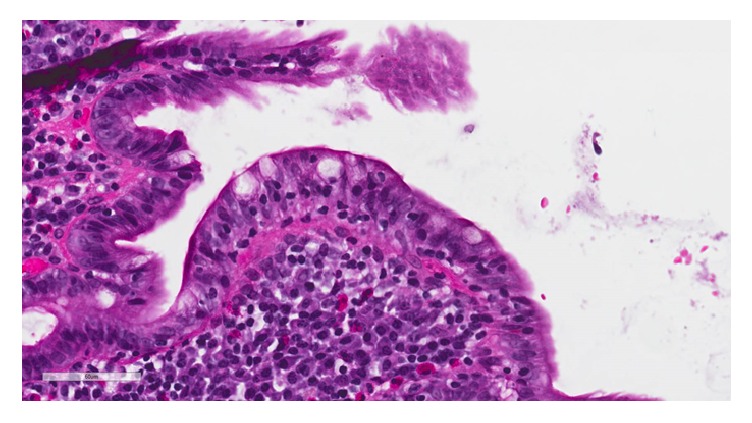
Higher power view highlighting a marked increase in intraepithelial lymphocytes.

Serologic studies for celiac disease revealed positive and significantly elevated antibodies: anti-gliadin IgA (>150 units, normal range < 20 units), anti-gliadin IgG (81.5 units, normal range < 20 units), anti-tissue transglutaminase IgA (127.3 units, normal range < 15 units), anti-tissue transglutaminase IgG (60.1, normal range < 15 units), and positive IgA endomysial antibodies (1 : 80 titer). Celiac associated HLA-DQ typing revealed a permissive genotype with positive HLA-DQ2 and HLA-DQ8. Esophagogastroduodenoscopy (EGD) showed nearly complete absence of duodenal folds along with flattened duodenal mucosa. Biopsies were taken and histology showed partial villous atrophy (villous : crypt ratio 1 : 3) with crypt hyperplasia and a marked increase in intraepithelial lymphocytes (80 lymphocytes per 100 epithelial cells) consistent with celiac sprue, Marsh classification 3B (Figures 2 and 3). Colonoscopy showed normal mucosa throughout the colon. He was started on a gluten-free diet and was prescribed iron supplements for 3 months. For his pulmonary embolism, he has been placed on long term anticoagulation using apixaban. Three months following the diagnosis, his hemoglobin, iron, and ferritin levels have normalized. He continues to follow a gluten-free diet and has experienced no adverse effects from anticoagulation.

## 3. Discussion

The majority of patients with celiac disease present with typical symptoms such as chronic diarrhea, failure to thrive, and weight loss. However, atypical and silent forms of the disease are being increasingly recognized, most likely due to better diagnostics, increased screening, and increased awareness of atypical presentations. In its atypical form, symptoms and findings of celiac disease are predominantly extraintestinal, while in its silent form symptoms and signs are completely absent [1, 4].

The most common hematologic manifestations of celiac disease are iron deficiency and megaloblastic anemia. Thrombocytosis or thrombocytopenia, IgA deficiency, and hyposplenism have also been described. Rare manifestations include venous thromboembolism, dermatitis herpetiformis, gluten ataxia, and celiac crisis syndrome [4, 5, 25]. The most feared, but thankfully rare, complications of untreated CD are enteropathy-associated T cell lymphoma and small bowel adenocarcinoma [1]. The diagnosis of celiac disease requires a high index of suspicion followed by screening for IgA anti-tissue transglutaminase antibodies (unless the patient has selective IgA deficiency in which case IgG anti-tissue transglutaminase antibodies should be measured) and is confirmed by small bowel biopsy [1, 26]. The prevalence of anemia in patients with celiac disease is estimated to be up to 20% [27]. Iron deficiency anemia is explained by decreased absorption of iron which is not surprising since the majority of iron is reabsorbed in the duodenum. IDA refractory to iron supplementation in the absence of bleeding should raise concern for CD [28, 29]. Folic acid is reabsorbed in the jejunum and folic acid deficiency is common in CD. Anemia secondary to folic acid deficiency is usually macrocytic. However, due to frequent and concomitant presence of IDA in patients with CD, macrocytosis is unusual [4]. Megaloblastic anemia due to deficiency of vitamin B12 is rare as vitamin B12 is mostly absorbed in the terminal ileum, which is usually spared in CD [30].

Several studies have investigated the association between CD and VTE [31–34]. These studies have yielded different and conflicting results. A cross-sectional study by Miehsler et al. [31] did not show increased risk of VTE among patients with CD; however, this study likely had low power since it was primarily designed to investigate risk of VTE among patients with inflammatory bowel disease, not CD. Two retrospective cohort studies by Ramagopalan et al. [32] and Zöller et al. [33] both showed statistically significant increased risk of VTE in patients with autoimmune disease, including celiac disease. A case control study from Denmark by Johannesdottir et al. [34] found significant risk of VTE only within 90 days from the diagnosis of celiac disease. Ludvigsson et al. [35] found a modest increase in risk of VTE in patients with celiac disease but that was restricted to individuals that were diagnosed in adulthood. They explained their findings based on a combination of surveillance bias and chronic inflammation. In an effort to summarize data and further investigate a potential association between celiac disease and the risk for VTE Ungprasert and colleagues from Mayo Clinic [36] conducted a meta-analysis that demonstrated significantly increased risk of VTE in patients with CD.

Over the last 30 years several case reports have been published suggesting a connection between CD and an increased risk of thrombosis and thromboembolism. We performed a literature search using PubMed for full articles, case reports, and case series published in English, involving adult patients, and using the following keywords, alone and in combination: “celiac disease”, “coeliac disease”, “gluten sensitive enteropathy”, “venous thromboembolism”, “deep venous thrombosis”, “pulmonary embolism”, “thromboembolism and celiac disease”, and “thrombosis and celiac disease”. Additionally, we added relevant studies and case reports from the reference list of selected articles. Cases without biopsy proven celiac disease were excluded. Our search yielded 19 cases (Table 1).

As we can see from Table 1 the most commonly reported location of thrombosis is in the hepatic veins (6/19) followed by DVT (5/19). Mesenteric vein thrombosis, portal vein thrombosis, and PE were reported in 3 cases each. Splenic vein thrombosis was reported in one case. Unusual places of thrombosis have been described such as LV thrombus in one case, central retinal vein thrombosis in one case, and two cases of cerebral vein thrombosis [7–24]. Among reported cases, patient median age was 33.1 years (range 18–53), with the majority of patients in the 4th decade of life (8/19). Of reported cases, 57% (11/19) were female patients. Most of the cases were reported from North Africa and the Middle East (9/19). The only case described from North America had central retinal vein thrombosis associated with celiac disease [14]

Three cases of pulmonary embolism have been described [7–9]. Interestingly, in the first case [9] the patient had celiac disease complicated by mesenteric vein thrombosis requiring surgery for mesenteric ischemia. Pulmonary embolism developed postoperatively; hence, CD alone cannot be solely responsible for the VTE and possibly perioperative risk was a contributing factor. The other two cases [7, 8] had elevated homocysteine levels, unlike in our patient, so it might be that hyperhomocysteinemia was the primary risk factor for VTE. Our patient interestingly did not have any of the traditionally recognized risk factors for VTE including elevated homocysteine level. In our case we argue that severe inflammation associated with celiac disease and evident by markedly elevated antibodies and biopsy findings were responsible for a procoagulant state and development of thrombosis.

DVT in patients with celiac disease has been described in 5 cases [7, 8, 18, 19, 22]. As mentioned above, in two of those cases the patients had elevated homocysteine levels; one case was associated with protein S deficiency; and in two cases [19, 22] similar to our case there were no other identifiable risk factors for thromboembolism apart from celiac disease. In 13 of 19 reported cases thromboses preceded the diagnosis of celiac disease and only in 7 of 19 cases did the patients present with classic symptoms of celiac disease. These are consistent with the previous statements suggesting that a significant number of patients with celiac disease have atypical or silent forms of the disease.

The mechanism by which celiac disease increases the risk of venous thromboembolism is unclear. However, several theories have been proposed:

(i)* Elevated homocysteine levels* secondary to folic acid deficiency due to malabsorption seen in celiac disease [37]. Saibeni et al. showed that the degree of intestinal injury correlates with homocysteine levels. However, this does not apply to our patient who had normal folic acid and homocysteine levels

(ii)* Elevated levels of thrombin-activatable fibrinolysis inhibitor (TAFI)* [38]

(iii)* Ongoing inflammation and provocative effect of inflammatory cytokines* secondary to the autoimmune nature of CD. It would be similar to other autoimmune diseases with well recognized increased risk for thrombosis (such as Crohn's disease and ulcerative colitis) [31]

(iv)* Protein C and S deficiency* secondary to vitamin K malabsorption [39]

(v)* Thrombocytosis leading to hyperviscosity of the blood* [9]. Thrombocytosis in celiac disease is likely secondary to IDA and/or hyposplenism. However, it cannot be solely responsible since not all patients with celiac disease have thrombocytosis. In reviewing the literature only 4 out of 12 patients who had a reported platelet value on admission had thrombocytosis

(vi)* Hyperviscosity secondary to high levels of circulating antibodies* including antiphospholipid antibodies [40]

(vii)* Genetic/environmental causes* since the majority of initial case reports were from North Africa and the Middle East (9 out of 19) [10, 23].

Extensive hypercoagulability work-up including screening for congenital or acquired prothrombotic disorders as well as malignancy in our patient failed to reveal any hypercoagulable disorders. Similarly, reviewing available cases published so far, only 6 cases out of 19 published had no other identifiable risk factor apart from celiac disease.

While there are clear guidelines on how to treat first and recurrent episodes of venous thromboembolism in the setting of provoked or idiopathic VTE, there are no guidelines for how long patients with celiac disease who develop VTE should be anticoagulated. Should we consider celiac disease a provoking factor that will disappear once GFD is started?

The majority of cases were treated with anticoagulation and all with GFD. However, duration of anticoagulation was not specified in many cases and greatly varied among those reported. For patients with recurrent thrombosis (cases 3, 19) lifelong warfarin was given. Cases with thrombosis despite GFD suggest that just GFD is not enough to completely eliminate the risk of thrombosis [23]. It is further supported by the study of Lee et al., who showed that the intestinal mucosa does not recover completely and inflammation does not completely resolve despite strict GFD up to 2 years following the diagnosis [41]. Interestingly, in one case [10] thrombosis reoccurred after stopping GFD despite anticoagulation which exemplifies the severity of inflammation in patients with CD who do not adhere to a gluten-free diet. Some authors suggest stopping anticoagulation once homocysteine levels return to normal [7]. It seems like a plausible solution in those cases where there is documented hyperhomocysteinemia, which might be a direct cause of VTE. However, in cases like ours, when levels of homocysteine are normal, it cannot be applied. Furthermore, the clinical trial by Den Heijer et al. [42] showed that homocysteine lowering by vitamin B and folic acid supplementation had no protective effect on the development of VTE.

We decided to anticoagulate our patient for 6 months using the direct oral anticoagulant apixaban, which has not been used before in this setting. We consider untreated celiac disease to be a provoking risk factor. We believe that even when adhering to a gluten-free diet, the inflammation and risk for VTE will not completely disappear; hence, following completion of anticoagulation we are planning on starting long term aspirin 81 mg daily. Should he experience recurrent DVT, he would need lifelong anticoagulation. At this moment it remains unclear whether we should use aspirin to prevent thrombotic complications in patients who are diagnosed with celiac disease, or at least shortly after the initial diagnosis when risk of VTE appears to be at its highest.

## 4. Conclusion

Here, we present a patient who developed extensive DVT and bilateral PE in the setting of untreated, silent celiac disease. Additionally, we summarized all similar case reports and reviewed the available literature on this topic. Due to better appreciation for atypical presentations of both celiac disease and venous thromboembolism, we expect that we will see more of these cases in the future. We will need more studies to determine the optimal duration of anticoagulation and more studies to determine the degree of VTE risk elevation in patients with CD.

## Figures and Tables

**Table 1:** Summary of published case reports on thrombosis associated with celiac disease.

Case [ref]	Year of publication	Age	Sex	Country	Site of thrombosis	Admission			GI symptoms on admission	Thrombosis risk factors	Order of occurrence	Comment	Treatment	Outcome
						Hgb	MCV	Plt						
1 [9]	1987	37	F	UK	Mesenteric vein, PE	n/a	n/a	654	Yes	Surgery	Thrombosis first	IgA deficiency + RF	GFD	Improved, without recurrence

2 [10]	1994	26	F	Algeria	Hepatic vein	126	85	153	Yes	None	CD first	Noncompliant with GFD	GFD	Lost in follow-up

3 [10]	1994	40	F	Algeria	Hepatic vein, PVT	120	69	450	Yes	OCP use, MPD	Thrombosis first	Interruption of GFD followed by thrombosis	GFD Long term Warfarin	Thrombosis reoccurred after stopping GFD despite anticoagulation

4 [11]	1995	46	F	Algeria	Hepatic vein, PVT	105	107	300	No	MPD	Thrombosis first	Low levels of folic acid and vit B12	GFD	Improved

5 [7]	1999	35	M	Australia	DVT, PE	n/a	n/a	n/a	No	Elevated homocysteine level, MTHFR mutation	Thrombosis first	Low folic acid	GFD Heparin Warfarin Folic acid	n/a Plan to stop warfarin if homocysteine level normalizes

6 [12]	2002	33	M	Israel	LV thrombus	10	71	n/a	Yes	Elevated homocysteine level, low folic acid level	Thrombosis first	Recurrent strokes in the setting of LV thrombus	GFD Warfarin Folic acid	Improved

7 [8]	2003	53	M	Italy	DVT, PE	108	n/a	n/a	No	Elevated homocysteine level, low folic acid level	Thrombosis first	Before DVT multiple prior episodes of thrombophlebitis	GFD Warfarin for 3 months	No recurrence in 1-year follow-up

8 [13]	2003	19	M	Spain	Hepatic vein	142	n/a	158	Yes	None	Thrombosis first	Had significant ascites, low folic acid	GFD Warfarin	No recurrence in 2-year follow-up

9 [14]	2005	33	F	USA	Central retinal vein	n/a	n/a	n/a	No	Pregnancy dehydration	CD first	Postpartum	Clopidogrel	n/a

10 [15]	2006	40	M	UK	Mesenteric veins	n/a	n/a	481	Yes	Protein S deficiency	CD first	Coexisting Crohn's disease	GFD	Improved

11 [16]	2006	30	F	Saudi Arabia	SMV, PVT, splenic vein thrombosis	73	n/a	518	No	Elevated homocysteine level	Thrombosis first	Failed iron supplementation	GFD Heparin Warfarin	Improved in 6 months' follow-up

12 [17]	2008	39	F	India	Splenic vein	11	n/a	170	No	Elevated homocysteine level	Thrombosis first	n/a	GFD LMWH	Improved in 6 months' follow-up

13 [18]	2009	18	M	Tunisia	DVT	135	n/a	440	No	Protein S deficiency	Thrombosis first	Significant weight gain with GFD	GFD Warfarin	Improved, symptom-free 4 years after diagnosis

14 [19]	2009	44	M	Turkey	DVT	11.7	n/a	n/a	Yes	None	CD first	n/a	LMWH Warfarin	Improvement

15 [20]	2009	19	M	India	Hepatic vein	6	n/a	175	No	Protein C and S deficiency	Thrombosis first	N/a	LMWH Warfarin	Improvement in 10 months' follow-up

16 [21]	2010	26	F	UK	Cerebral vein thrombosis	n/a	n/a	n/a	No	None	CD first	Lane Hamilton syndrome	UFH	Improvement

17 [22]	2012	30	F	Iraq	DVT	5	n/a	950	No	None	Thrombosis first	n/a	GFD Warfarin	Improvement

18 [23]	2016	27	F	Jordan	Hepatic vein	n/a	n/a	145	No	None	CD first	Thrombosis occurred despite compliance with GFD	Warfarin portocaval shunt for ascites	Improved

19 [24]	2017	35	F	Tunisia	Cerebral vein thrombosis	9.4	n/a	n/a	No	OCP use	Thrombosis first	Recurrent cerebral vein thrombosis upon cessation of AC	Warfarin	Improvement

Hgb: hemoglobin (g/L), MCV: mean corpuscular volume, PLT: platelets (10^9^/L), CD: celiac disease, DVT: deep venous thrombosis, OCP: oral contraceptive, GFD: gluten-free diet, PVT: portal vein thrombosis, AC: anticoagulation, SMV: superior mesenteric vein, LV: left ventricle, LMWH: low molecular weight heparin, and MTHFR: methyl-tetrahydrofolate reductase.
